# Attentional lapses are reduced by repeated stimuli having own-name during a monotonous task

**DOI:** 10.1371/journal.pone.0194065

**Published:** 2018-03-07

**Authors:** Kosuke Kaida, Takashi Abe

**Affiliations:** 1 Automotive Human Factors Research Center, National Institute of Advanced Industrial Science and Technology (AIST), Tsukuba, Ibaraki, Japan; 2 International Institute for Integrative Sleep Medicine (WPI-IIIS), University of Tsukuba, Ibaraki, Japan; Boston Children’s Hospital / Harvard Medical School, UNITED STATES

## Abstract

The goal of the present study was to examine the effect of listening to self-relevant words (i.e., one’s own name) on vigilant attention, arousal, and subjective sleepiness during performance of a psychomotor vigilance test (PVT). Twenty-one participants aged 20–26 years (22.2 ± 1.76) performed a PVT in four experimental conditions: one in which their own full name was pronounced every 20 s in the stimuli epochs, one in which their full name was pronounced in inverted form, one in which beeps were played, and a control condition with no stimuli. Listening to personal names reduced attentional lapses during the PVT (i.e., the number of reaction times no less than 500 ms). The results are a first step in applying the name effect to technologies and devices aimed at maintaining arousal levels and preventing accidents during a monotonous task, such as driving.

## Introduction

Performance deterioration due to sleepiness has been considered to be one of the major causes of work-related injuries and traffic accidents [1, 2]. A variety of countermeasures have been tested to reduce sleepiness and prevent accidents; these have included listening to music [3], chewing gum [4], and exposure to cool air [5]and light [6, 7]. Most researchers agree that there are only a few practical ways to reduce sleepiness, such as taking a nap [8, 9] and ingesting caffeine [10–12]. While naps and caffeine are effective in reducing sleepiness and improving cognitive performance, drivers often report that conversation can also help them resist dropping off at the wheel [13, 14]. Previous studies have indeed reported that conversation can be effective in reducing sleepiness [15–17].

In the present study, we focused on the arousal factor of self-relevant information during a conversation. Self-relevant information, such as the names of family members, close friends, and oneself, as well as sounds involving personal concerns, has been known to be a strong attractor of human attention [18]. In nature, humans automatically focus their attention on self-relevant information to maximize benefits and prevent loss of opportunities, a phenomenon captured in the so-called cocktail party effect [19, 20]. A person’s own name particularly draws automatic attention, probably because it is the first lexical item that humans learn [21]. This is likely an effect of classical conditioning, in which humans learn their own names because they always occur during events related to the self. As a result of experiencing countless references to one’s name in self-relevant events throughout life, one’s own name comes to have a special value as a conditioned stimulus. Such a spontaneous relationship should also automatically induce a conditioned response, such as an increase in arousal level.

The occurrence of one’s name has indeed been shown to increase arousal level, as measured by event-related potential (ERP; [22–24]). This suggests that paying attention to a salient stimulus automatically should increase arousal. Because arousal is known to activate broad areas of the brain [25, 26], vigilant attention should also improve when the occurrence of a person’s own name (i.e., self-relevant information) increases arousal. Thus, an increase in arousal triggered by one’s own name could be a fundamental contributor to improved performance on a vigilance task.

We used the psychomotor vigilance test (PVT) to measure performance. In the PVT, reaction times and lapses are known to be sensitive indices of sleep deprivation [27]. Moreover, performance in the PVT is well correlated with subjective sleepiness and alpha (8.0–12.0 Hz) and theta (4.0–7.9 Hz) band spectral power density of the electroencephalogram (EEG) [28]. Estimating the arousal level using EEG has advantages such as the real-time estimation of subjective sleepiness during task performance, which cannot be assessed using verbal responses because verbal rating affects sleepiness [17].

The goal of the present study was to examine the effect of self-relevant auditory information (i.e., one’s own name) on arousal level and performance. We hypothesized that hearing one’s own name would increase arousal compared to listening to stimuli that are irrelevant to the self. These self-relevant stimuli should contribute to maintaining performance in a monotonous task, such as a psychomotor vigilance test [27], in which performance is usually impaired when participants are bored.

## Methods

### Participants and design

Participants were 21 healthy, native Japanese speakers aged 20–26 years (22.2 ± 1.76, 6 female). Participants were recruited through the website of the author’s laboratory. The experiment was conducted between February and July of 2017. All participants met the following criteria: (1) a normal sleep-wake cycle, classified as intermediate type according to the Morningness–Eveningness questionnaire [29, 30], (2) no report of any physical or mental health problems, and a score of no less than 15 on the Center for Epidemiological Studies-Depression Scale (CES-D; [31, 32], (3) no experience of shift work within the 3-month prior to the experiment, (4) no travel to a different time zone within the 3-month prior to the experiment, (5) no use of any medication, (6) no use of tobacco products, and (7) a body mass index less than 25 (calculated as weight in kilograms divided by the square of the height in meters; BMI). Participants’ scores (mean ± standard deviation) were as follows: ME, 47.6 ± 8.16; CES-D, 10.7 ± 5.75; and BMI, 20.7 ± 3.01 kg/m^2^. Participants reported that they had slept for 494.3 ± 99.78 min on the previous night of the day of the experiment. All were paid for their participation.

**Fig 1:**
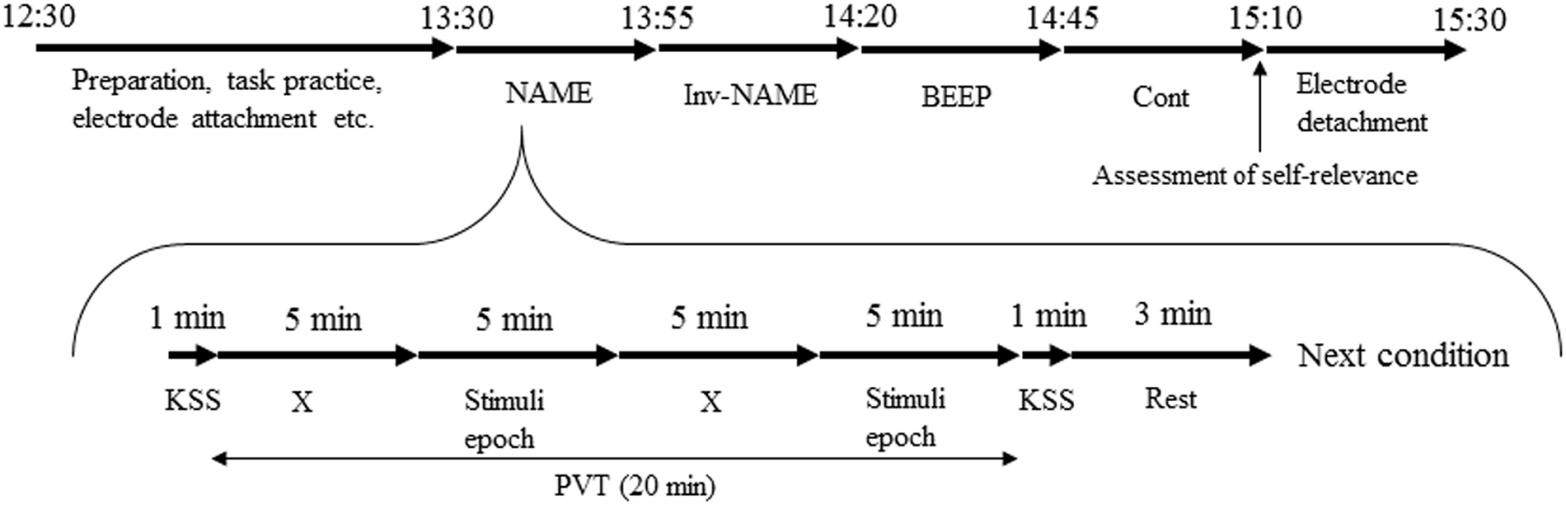
Schedule of the experiment. NAME: name condition, Inv-NAME: inverted name condition, BEEP: beep condition. Cont: control condition, KSS: Karolinska Sleepiness Scale, PVT: psychomotor vigilance test. In the stimuli epochs, a stimulus was presented every 20 s. The order of the condition was counterbalanced among the participants.

Participants arrived at the laboratory at 12:30, and, after receiving a full explanation of the procedure, signed an informed consent document. We confirmed that all the participants had eaten lunch before arriving at the lab. The experiment began at 13:15 after electrodes for EEG and electrooculogram (EOG) were attached. The schedule of the experiment is shown in Fig 1.

In one test session, participants carried out a PVT [27] for 20 min without breaks (see Fig 1). During the first 0–5 min of the PVT session, a no stimuli epoch, participants carried out the task in silence. In the next 5–10 min, a stimuli epoch, participants were exposed to an auditory stimulus every 20 s or remained in a silent condition. The following 10–15 min was another no stimuli epoch, and the final 15–20 min was a stimuli epoch of the same type as the first stimuli epoch. Participants carried out four test sessions to complete all the experimental conditions, which took them around 80 minutes in total. Participants were allowed to take a short break after each 20 min session if needed.

Participants took part in all of the following four experimental conditions (within-subject experimental design), with the order of conditions counterbalanced among participants: (1) a name condition (NAME), (2) an inverted name condition (Inv-NAME), (3) a beep condition (BEEP), and (4) a no stimuli control condition (Cont). In the NAME condition, the participant’s own full name was played every 20 s in the stimuli epochs. In the Inv-NAME condition, participants heard the sounds of their full name played in inverted form every 20 s in the stimuli epochs. In the Japanese language, it is always possible to invert and pronounce the syllables of a name, because any word in the language can be decomposed into a sequence of vowels alone or combinations of consonant(s) and vowel(s), which can then be inverted and read aloud. For example, the sequence of consonants and vowels in the name *Akira Kurosawa* (A-KI-RA-KU-RO-SA-WA) can be inverted and read as Wasaroku Rakia (WA-SA-RO-KU-RA-KI-A). To a native Japanese speaker, the inverted name sounds very different from the original name and thus is not associated with the self. Thus, the inverted presentation of personal names as stimuli in the Inv-NAME condition maintained the identical combination of vowels and consonants as that in the NAME condition, but in a different order. Using this inversion technique, we were able to create an Inv-NAME stimulus for each participant that was unique but consistently designed in terms of self-relevance. In the BEEP condition, a 1000 Hz pure tone was used as the stimuli. In the Cont condition, no stimuli were presented during the 20-min task. The stimuli in both NAME and Inv-NAME conditions were created using artificial text reading software (Textalk, ver. 1.0.0.1, Wity Co., Ltd., Japan) with a female voice tone. Each stimulus lasted around 2 s (depending on the length of the full name). Sound pressure level was set at 70 dB measured at the ear level.

Ethical considerations of the experimental protocol were reviewed and approved by the review board at the National Institute of Advanced Industrial Science and Technology (AIST) of Japan, according to the principles expressed in the Declaration of Helsinki.

### Electroencephalogram (EEG)

Electrodes were attached at the Cz scalp site for EEG referenced to linked electrodes at the earlobes, and outside both canthi for EOG. The sampling rate was 1000 Hz (24-bit AD conversion), and the time constants were 0.3 s for the EEG and 3.2 s for the EOG. Electrode impedance was maintained below 5 kΩ. The low-pass filter was set at 30 Hz. Electrophysiological data were recorded with a portable digital recorder (PolymateV AP5148, Digitex Laboratory Co., Ltd., Japan).

Alpha (8.0–12.0 Hz) and theta (4.0–7.9 Hz) power spectra during PVT were calculated using fast Fourier transform (FFT; frequency resolution, 0.97 Hz) with a Hamming window. FFT was performed using the data of each stimulus epoch (i.e., 5–10 and 15–20 min epochs from the start). The data of the first 60 s in each epoch were omitted to avoid startle responses. In total, the data of 270 s in the stimuli epochs were used for FFT analysis by commercial software (CSA Sleep Analysis, version 1.16, NoruPro Light Systems, Inc., Japan). FFT was applied to overlapping (by 0.024 s) EEG segments of 1.024 s and was subsequently averaged for one 240 s epoch. Electromyogram and electrooculogram in the EEG were reduced using high-pass (0.5 Hz) and low-pass (30 Hz) digital filters.

### Psychomotor vigilance test (PVT)

The PVT uses a visual reaction time paradigm with interstimulus intervals ranging from 2 to 10 s [33]. Participants were instructed to monitor a red square shown in the middle of the computer display and press a response button on the keyboard as soon as a yellow number counting down by milliseconds appeared within the square. The counter stopped upon the participant’s response, and the reaction time in milliseconds was displayed for one second period as feedback to the participant. Responses within 100 ms received warning signals (“FS”; false start) for one second period. No response within 30 s after the counter started also received a warning signal (“OVERRUN”) for one second period. Both the False Starts and Overruns were treated as timeout trials, which continued to the next trial. The PVT was programmed using E-prime version 1.2 (Psychology Software Tools, Inc.).

As noted above, the data of the first 60 s in each epoch were omitted. Means of the reciprocal reaction times (RRT) and the number of lapses, i.e. responses no less than 500 ms [33, 34], were calculated as performance indices. The data from the two stimuli epochs in each condition were averaged to obtain a stable data set. The data of two no-stimuli epochs in each condition were also averaged.

### Rated sleepiness

The 9-point Karolinska Sleepiness Scale [28, 35] was used by participants to rate their degree of sleepiness on a scale that included 1 (*very alert*), 3 (*alert*), 5 (*neither alert nor sleepy*), 7 (*sleepy*, *but not fighting sleep*), and 9 (*very sleepy*, *fighting sleep*). Participants were asked to circle the number that represented their personal feeling at that moment. The KSS was administered twice, at the beginning and end of the PVT.

### Rated self-relevance to the stimuli

Participants were asked to evaluate the stimuli presented in the stimuli epochs using a 9-point scale that included 1 (*very relevant*), 3 (*relevant*), 5 (*neither relevant nor irrelevant*), 7 (*irrelevant*), and 9 (*utterly irrelevant*). Ratings of self-relevance of the stimuli were made at the end of each PVT block (i.e., every 20 min).

### Statistical analysis

Linear mixed models were employed for statistical analyses of the present data. The restricted maximum likelihood was used as the estimation method. The condition was modeled as a fixed effect, and the participant was modeled as a random effect. A variance component was used for the covariance structure of the random effect. For post-hoc analysis, the data were calculated as values subtracted from the Cont condition. Differences from zero in the NAME, Inv-NAME, and BEEP conditions were tested using paired one sample *t*-tests. Paired *t*-tests were employed to compare the self-relevance scores of the stimuli of the three conditions. All statistical analyses were performed using the SPSS system for Windows, version 22.0.

## Results

### Self-relevance of the stimuli

**Fig 2:**
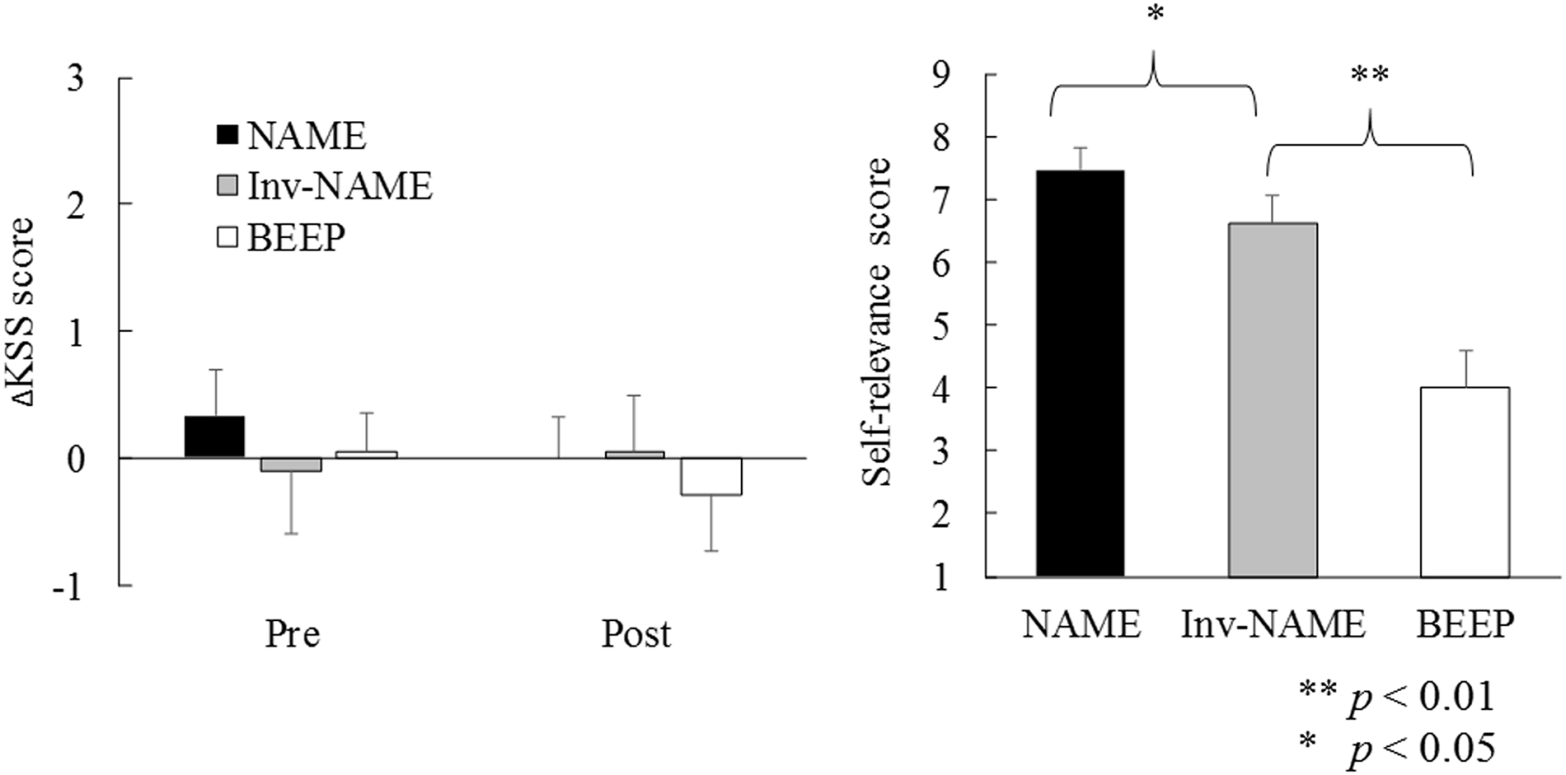
Subjective sleepiness (KSS) scores (left) and self-relevance scores for the stimuli (right). NAME: name condition, Inv-NAME: inverted name condition, BEEP: beep condition. Cont: control condition, KSS: Karolinska Sleepiness Scale.

The main effect of condition on self-relevance was significant, *F* (2, 60) = 13.92, *p* < 0.01. Self-relevance scores were highest in the own name condition: NAME vs. BEEP conditions, *t* (20) = 4.36, *p* < 0.01; NAME vs. Inv-NAME conditions; *t* (20) = 2.26 *p* < 0.05; see Fig 2, right panel. Scores for inverted personal name stimuli were also higher than those for beep stimuli: Inv-NAME vs. BEEP conditions, *t* (20) = 3.05, *p* < 0.01.

### PVT

The main effect of condition in the number of lapses was marginally significant, *F* (3, 60) = 2.20, *p* = 0.09. The number of lapses was significantly smaller in the NAME condition than in the Cont condition, *t* (20) = 2.20, *p* < 0.05. The Inv-NAME and BEEP conditions were not significantly different compared to the Cont condition, *t* (20) = 0.15, *p* = 0.87 and *t* (20) = 0.72, *p* = 0.47, respectively.

**Fig 3:**
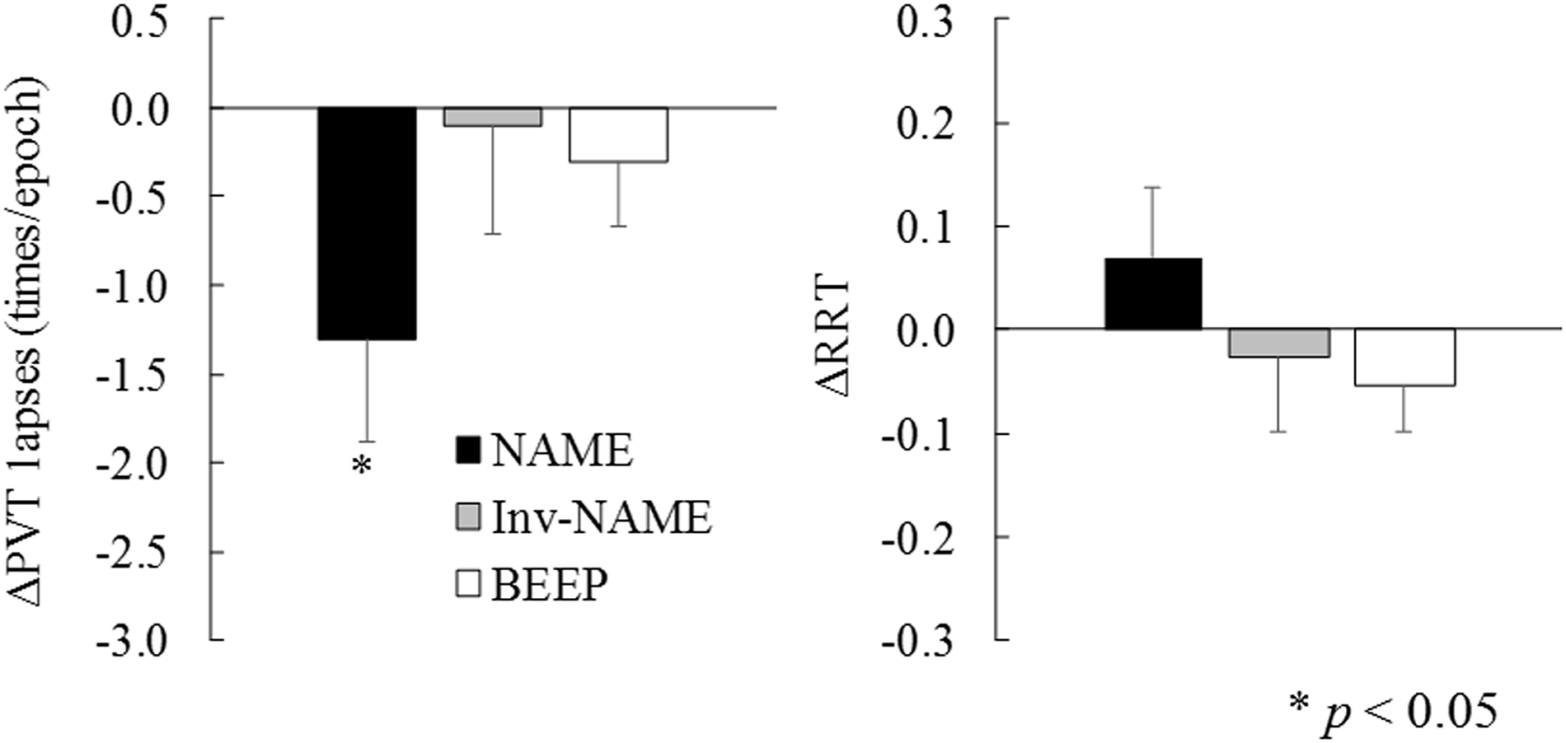
Lapses (left) and reaction times (right) in the psychomotor vigilance test. NAME: name condition, Inv-NAME: inverted name condition, BEEP: beep condition. Cont: control condition, PVT: psychomotor vigilance test, RRT: reciprocal reaction time. The data shown are subtracted values from the Cont condition.

The main effect of condition in the RRT was not significant (*F* (3, 60) = 1.23, *p* = 0.30). As shown in Fig 3, the RRT were not significant in any condition compared to the Cont condition (*t* (20) = 1.03, *p* = 0.32; *t* (20) = 0.38, *p* = 0.71; *t* (20) = 1.12, *p* = 0.24 for NAME, Inv-NAME, BEEP conditions, respectively).

### Spectral power density of EEG

The main effect of condition on EEG spectral power density was not significant (*F* (3, 60) = 1.08, *p* = 0.36). However, in the post hoc analysis, a tendency emerged for alpha power density to be lower in the NAME condition than in the Cont condition, *t* (20) = 1.93, *p* = 0.06. Alpha power densities did not differ from the Cont condition in the Inv-NAME condition, *t* (20) = 1.27, *p* = 0.21, or in the BEEP condition, *t* (20) = 0.94, *p* = 0.35. These results suggest that arousal level during the stimuli epochs tended to be better maintained in the Name condition than in the Cont condition.

**Fig 4:**
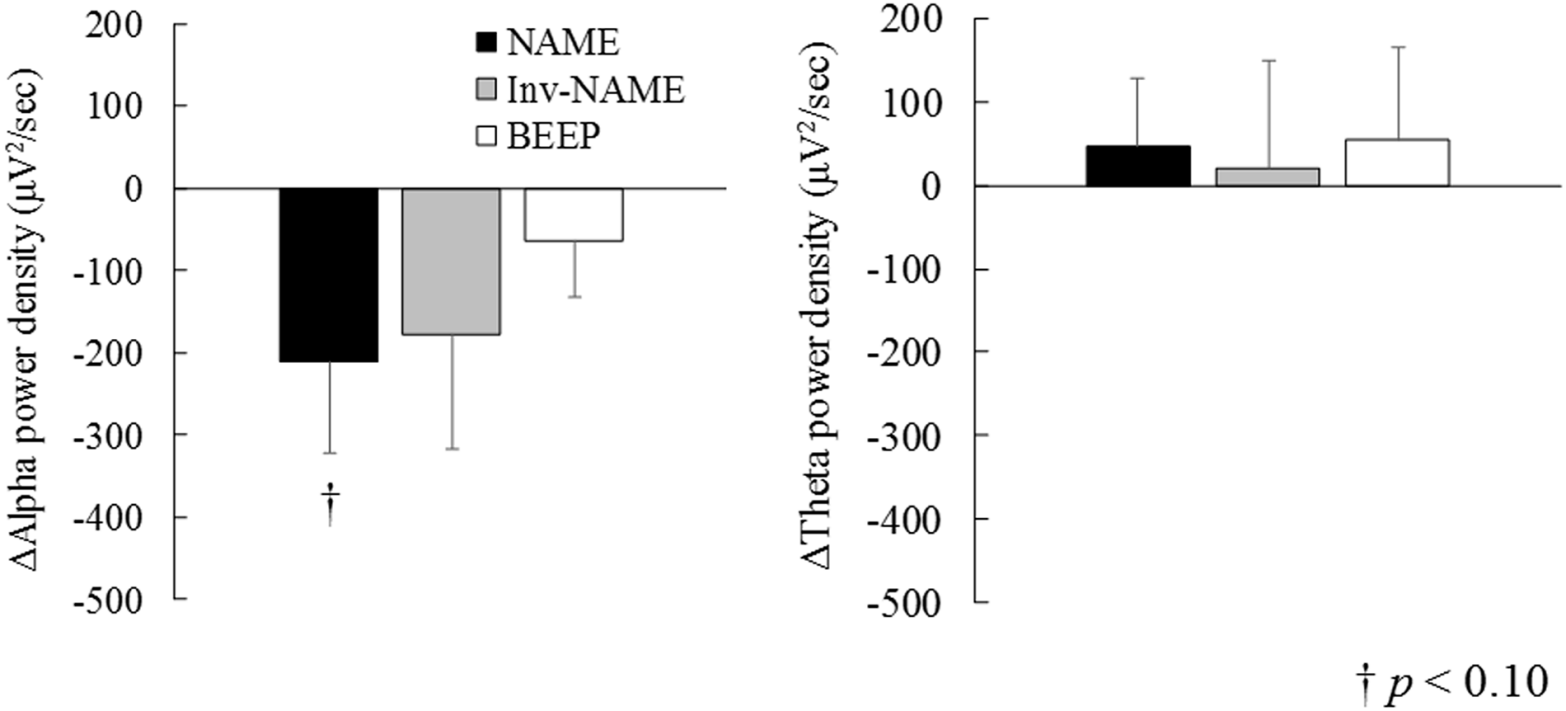
Spectral power densities in alpha (left) and theta (right) band frequencies. NAME: name condition, Inv-NAME: inverted name condition, BEEP: beep condition. Cont: control condition, KSS: Karolinska Sleepiness Scale. The data shown are subtracted values from the Cont condition.

As for theta power densities, there were no significant differences detected in the analysis of linear mixed models, *F* (3, 60) = 0.08, *p* = 0.96, and the post-hoc analysis compared to the Cont condition (*t* (20) = 0.58, *p* = 0.56; *t* (20) = 0.15, *p* = 0.87; *t* (20) = 0.48, *p* = 0.63 for the NAME, Inv-NAME, BEEP conditions, respectively). These results are shown in Fig 4.

### Subjective sleepiness

There were no significant differences in subjective sleepiness in the mixed model analysis, either before or after the task, *F* (3, 60) = 0.37, *p* = 0.77. Post-hoc analyses among the conditions also produced no significant differences before or after the task, *t* (20) < 1 for all comparisons. These results are shown in Fig 2 (left panel).

## Discussion

The present results suggest that the presentation of a personal name can be effective in maintaining arousal and preventing attentional lapses during monotonous tasks. Hearing one’s own name affected performance and arousal level during a vigilance task by reducing attentional lapses and alpha power density in the EEG. While effects of personal names on arousal (i.e., the “name effect”; [36] have been previously reported [22–23], to the best of our knowledge, the present study provides the first evidence of such effects on attentional lapses.

The present results indicate that the auditory presentation of a personal name has a special impact on attentional lapses in a monotonous task. This finding regarding the name effect on attentional lapses is straightforward but is potentially meaningful for future applications. For example, a sleepiness detection device would be more effective in alerting (i.e., giving feedback) and in keeping drivers awake by calling their own names upon detecting their drowsiness, compared to merely emitting a beeping sound. Although the effect may last only for a short period, it might be sufficient for drivers to safely reach a rest area to take a break for caffeine or to take a nap for attentional recovery.

Previous studies have reported that greater amounts of self-relevant information elicit larger event-related potentials (ERP), such as P3, both during awake periods [22–24] and sleep periods [37]. It is also known that EEG alpha density is well correlated with subjective sleepiness [38, 39] and attentional lapses in PVT [28]. One advantage of using EEG alpha density is that it is easier to measure and calculate than ERP. The utility of EEG alpha in relation to attentional lapses was confirmed here.

Given the present results, we believe that a personal name is a good stimulant to maintain arousal and prevent attentional lapses during a monotonous task. However, the name effect was not detected in subjective sleepiness. There are two possible reasons for this. First, the name effect may have been too marginal to remain after the task was finished. The participants evaluated not their sleepiness during the task but their *current* sleepiness, that is, after the stimuli epoch. This procedure may have produced a null effect for subjective sleepiness while confirming the effect on task performance. Second, the accuracy of self-evaluations of sleepiness may have been inadequate. It is known that behavioral, physiological, and subjective indices of sleepiness are sometimes disassociated from each other, especially under stimulus conditions [40, 41]. Because subjective sleepiness data can be sensitively affected by the rating environment, it should be collected in carefully designed experimental conditions.

The functional mechanism of the name effect is not yet fully understood. The name effect could be caused by stimulating the neuromodulatory brain systems that produce general excitability of cortical neurons. Stimulation of these systems modulates vigilant attention and constitutes a precondition to respond to stimuli [25, 42–44]. This precondition should increase arousal and decrease mind wandering that often occurs when we carry out a monotonous and boring task.

Mind wandering is known to be a cause of attentional lapses [45]. It has been reported that listening to self-related information concerning worry and anxiety induces mind wandering because it plays the role of priming thoughts about negative issues [46]. Therefore, such self-related stimuli might increase attentional lapses instead of reducing them. To avoid the mind wandering aspect of self-relevance, more neutral stimuli such as a person’s name, the inverted name, and a pure tone, which are not related to worry and anxiety and was not expected to induce mind wandering were used in this study. As a result, it was expected that any observed attentional lapses would be unrelated to the potential influence of negative valance and demonstrate the self-relevancy effect.

Lapses are not only caused by attentional dysfunctions, but also by behavioral “microsleep”, which is a short period of disengagement from a task lasting approximately 3 s [47] that can occur even in non-sleep-deprived individuals [48]. Hearing one’s name might reduce microsleep. Moreover, the reduction of microsleep could be reflected in a reduction of lapses in the PVT. The causal mechanism of the name effect remains an interesting topic for future studies.

Further investigations of name effect, or self-relevant effects, in other sensory modalities, such as smell, touch, taste, and sight, are also warranted. Effects of the sense of sight have been reported, with visually presented personal names eliciting larger ERPs than no self-relevant stimuli [36]. As far as we know, similar effects for smell, touch, and taste have not been reported. Presentation in multi-dimensional modalities may produce stronger and more preferable effects on arousal and attentional lapses.

In the present study, we confirmed that both vigilance task performance and subjective self-relevance were significantly greater in the NAME condition compared to the other three conditions. However, for the vigilance task, we did not find any significant differences between the Inv-NAME condition and the other two less self-relevant conditions of BEEP and Cont, although subjective self-relevance was significantly higher in the Inv-NAME condition than in the latter two. Thus, we could not clarify the strength of the relevance effect according to the degree of self-relevance. We assumed that the objective (i.e., measured by task performance) name effect would reflect the degree of self-relevance of the stimuli as recognized by individuals. Clarifying this point remains for future investigation.

In conclusion, the present study demonstrated the effectiveness of using personal names as self-relevant information on increasing arousal levels and preventing attentional lapses during a monotonous task. The findings provide a first step in applying the name effect to technologies and devices intended to maintain arousal and prevent accidents during monotonous tasks such as driving.
